# Renal Function Impact in the Prognostic Value of Galectin-3 in Acute Heart Failure

**DOI:** 10.3389/fcvm.2022.861651

**Published:** 2022-04-07

**Authors:** Pedro Caravaca Perez, José R. González-Juanatey, Jorge Nuche, Lucia Matute-Blanco, Isabel Serrano, Manuel Martínez Selles, Rafael Vázquez García, Luis Martínez Dolz, Manuel Gómez-Bueno, Domingo Pascual Figal, María G. Crespo-Leiro, Álvaro García-Osuna, Jordi Ordoñez-Llanos, Juan Cinca Cuscullola, José M. Guerra, Juan F. Delgado

**Affiliations:** ^1^CIBER de Enfermedades Cardiovasculares (CIBERCV), Madrid, Spain; ^2^Department of Cardiology, Hospital Universitario 12 Octubre, Instituto de Investigación Sanitaria Hospital 12 Octubre (Imas12), Madrid, Spain; ^3^Department of Cardiology, Facultad de Medicina, Complejo Hospitalario Universitario de Santiago de Compostela, Universidad de Santiago, Santiago de Compostela, Spain; ^4^Centro Nacional de Investigaciones Cardiovasculares, Madrid, Spain; ^5^Department of Cardiology, Hospital Universitari Arnau de Vilanova, IBRLLEIDA, Lleida, Spain; ^6^Department of Cardiology, Hospital Universitario Joan XXIII, Instituto de Investigación Sanitaria Pere Virgili, Universidad Rovira i Virgili, Tarragona, Spain; ^7^Department of Cardiology, Hospital Universitario Gregorio Marañón, Instituto de Investigación Sanitaria IiGM, Universidad Europea, Madrid, Spain; ^8^Department of Cardiology, Hospital Universitario Puerta del Mar, Cádiz, Spain; ^9^Departamento de Medicina, Universidad de Cádiz, Instituto de Investigación e Innovación Biomédica de Cádiz, Cádiz, Spain; ^10^Department of Cardiology, Hospital Universitario y Politécnico La Fe, IIS La Fe, Valencia, Spain; ^11^Department of Cardiology, Hospital Universitario Puerta de Hierro, Majadahonda, Spain; ^12^Department of Cardiology, Hospital Universitario Virgen de la Arrixaca, Universidad de Murcia, Murcia, Spain; ^13^Department of Cardiology, Complexo Hospitalario Universitario A Coruña, Instituto de Investigación Biomedica A Coruña, Universidade da Coruña, A Coruña, Spain; ^14^Servicio de Bioquímica-IIB Sant Pau, Departamento de Bioquímica y Biología Molecular, Universitat Autónoma, Barcelona, Spain; ^15^Fundación Para la Bioquímica y la Patología Molecular, Barcelona, Spain; ^16^Department of Cardiology, Hospital Universitario Santa Creu i Sant Pau, IIB Sant Pau Universitat Autónoma de Barcelona, Barcelona, Spain; ^17^Facultad de Medicina, Universidad Complutense de Madrid, Madrid, Spain

**Keywords:** acute heart failure (AHF), galectin-3 (Gal-3), cardiorenal syndrome (CRS), renal function, prognosis

## Abstract

**Introduction:**

Galectin-3 (Gal-3) is an inflammatory marker associated with the development and progression of heart failure (HF). A close relationship between Gal-3 levels and renal function has been observed, but data on their interaction in patients with acute HF (AHF) are scarce. We aim to assess the prognostic relationship between renal function and Gal-3 during an AHF episode.

**Materials and Methods:**

This is an observational, prospective, multicenter registry of patients hospitalized for AHF. Patients were divided into two groups according to estimated glomerular filtration rate (eGFR): preserved renal function (eGFR ≥ 60 mL/min/1.73 m^2^) and renal dysfunction (eGFR <60 mL/min/1.73 m^2^). Cox regression analysis was performed to evaluate the association between Gal-3 and 12-month mortality.

**Results:**

We included 1,201 patients in whom Gal-3 values were assessed at admission. The median value of Gal-3 in our population was 23.2 ng/mL (17.3–32.1). Gal-3 showed a negative correlation with eGFR (rho = −0.51; *p* < 0.001). Gal-3 concentrations were associated with higher mortality risk in the multivariate analysis after adjusting for eGFR and other prognostic variables [HR = 1.010 (95%-CI: 1.001–1.018); *p* = 0.038]. However, the prognostic value of Gal-3 was restricted to patients with renal dysfunction [HR = 1.010 (95%-CI: 1.001–1.019), *p* = 0.033] with optimal cutoff point of 31.5 ng/mL, with no prognostic value in the group with preserved renal function [HR = 0.990 (95%-CI: 0.964–1.017); *p* = 0.472].

**Conclusions:**

Gal-3 is a marker of high mortality in patients with acute HF and renal dysfunction. Renal function influences the prognostic value of Gal-3 levels, which should be adjusted by eGFR for a correct interpretation.

## Introduction

Chronic kidney disease (CKD) is a common comorbidity in patients with acute heart failure (AHF), complicating clinical management and worsening patient's prognosis (1). To better know the cardiorenal interaction in patients with AHF, biomarkers assessing pathophysiological pathways and improving risk stratification are needed.

Galectin-3 (Gal-3) is a lectin, a member of the beta-galactoside-binding protein family, involved in processes of oxidative stress, inflammation and cellular fibrosis (2). In patients with heart failure (HF), Gal-3 is a valuable biomarker provided its role in the development of myocardial damage, cardiac remodeling and further progression of the disease (3, 4). Likewise, Gal-3 is a key mediator in developing fibrosis in multiple renal disorders associated with the progressive worsening of renal function (5). Due to this relationship with inflammation and fibrosis processes at the cardiac and renal levels, Gal-3 is postulated as a promising biomarker in the setting of cardiorenal syndrome (6).

However, the prognostic value of Gal-3 in relation to the different stages of the renal disease in patients with AHF is not well known (7). This could be useful, not only for risk assessment but for selecting candidates that would benefit from specific treatment strategies. This work aims to evaluate the prognostic interaction between the levels of Gal-3 and the renal function impairment in patients admitted for AHF.

## Materials and Methods

### Study Population and Data Collection

The Redinscor-II is an observational, prospective, multicenter completed between October 2013 and December 2014, in 20 university hospitals in Spain (8). A total of 1,831 patients ≥ 18-year-old, with symptoms and signs of AHF (either *de novo* or chronic decompensated) and requiring hospitalization, were included. Diagnosis and treatment AHF episode was conducted by a specialist in cardiology following the recommendations of the current HF guidelines (9).

Patients with HF in the context of ST-segment elevation acute coronary syndrome; those whose vital prognosis was <1 year; and those unable to fulfill outpatient follow-up were excluded. After discharge, all patients underwent follow-up for events assessment at 1, 3, 6, and 12 months after inclusion.

The study had the approval of the ethics committees of all the participating hospitals. All included patients signed the informed consent.

For every patient included in the registry, all demographic and clinical variables were recorded during hospitalization. For this analysis, we only included patients in whom on-admission serum Gal-3 concentrations had been quantified.

Plasma creatinine was used to calculate the estimated glomerular filtration rate (eGFR) according to the CKD-Epidemiology Collaboration (CKD-EPI) formula. Renal failure on admission was as defined as an eGFR <60 mL/min/1.73 m^2^. Patients were divided into 4 groups, according to the presence or absence of renal dysfunction and Gal-3 concentrations above/below the median for each group. Thus, the 4 groups were defined as follows:

- Preserved renal function and Gal-3 ≤ 19 ng/mL
- Preserved renal function and Gal-3 > 19 ng/mL
- Renal dysfunction and Gal-3 ≤ 28.2. ng/mL
- Renal dysfunction and Gal-3 > 28.2 ng/mL.

### Biomarkers Measurement

Blood samples were obtained in the first 24 h of admission. They were centrifuged at room temperature at 2.500 g for 15 min. Aliquots of serum and heparinized or EDTA-anticoagulated plasma were frozen at −80°C until analysis. Serum Gal-3 concentrations were assessed in a core-lab laboratory (Hospital de la Santa Creu i Sant Pau, Barcelona), by immunoassay on a Mini-Vidas platform (BioMérieux, Marcy-l'Étoile, France). The employed immunoassay detects Gal-3 in a range between 3.3 and 100 ng/mL with an imprecision <5.5%. The imprecision observed in the central laboratory was equal to or lower than that reported by the manufacturer. Concentrations of aminoterminal portion of proBNP molecule (NT-proBNP) were also quantified in serum in the core laboratory. Personnel responsible for Gal-3 and NT-proBNP assessment were blind to the patients' clinical data.

### Statistical Analysis

Quantitative variables were expressed as mean and standard deviation (SD) or median and interquartile range (IQR, expressed as 25th percentile−75th percentile). Qualitative variables as absolute and relative frequency. The chi-square test or Fisher's exact test were used to compare qualitative variables; and ANOVA or Kruskal-Wallis test, and Student's *t*-test or Mann-Whitney *U*-test, when appropriate, to compare quantitative variables. Multivariate linear regression analysis was performed, after logarithmic transformation, to evaluate the predictors that were associated with increased Gal-3 concentrations, reported as the beta coefficient. Variables were eliminated from the initial model using the backward elimination method with a cutoff point of *p* < 0.05.

The main outcome was all-cause mortality at 12-month follow-up. The Kaplan-Meier method was used to study the association between Gal-3 values and mortality. The different survival curves were compared by log-rank test. To identify predictors of the 12-month mortality occurrence during the 12-month follow-up, a stepwise Cox regression analysis was performed to develop a model to estimate the effect of Gal-3 on the main event. First, variables with univariate association with mortality with a *p*-value < 0.1 were identified and included as candidate variables in the initial model. The main covariates included in the model were age, diabetes mellitus, previous diagnosis of HF, presence of prior admissions for HF, blood pressure, hemoglobin, natremia, eGFR, NT-proBNP, left ventricular ejection fraction (LVEF), presence of congestive signs at discharge, NYHA functional class, treatment at discharge with renin-angiotensin-aldosterone system inhibitors and beta-blockers. Finally, to make a simpler model without affecting the estimation of the effect, variables were eliminated from the initial model using the backward elimination method with a cutoff point of *p* < 0.05. The proportional hazard assumption was evaluated using the Schoenfeld residuals test. The discriminative ability of the models was estimated by Harrell's C statistic and calibrated by the Groennesby and Borgan test. Restricted cubic spline curve was used to display the association of Gal-3, as a continuous variable, and the hazard ratio for all-cause mortality, using three knots.

Diagnostic performance curves (ROC curves) were used to study the optimal cutoff points of Gal-3 for predicting mortality within eGFR groups. Youden's index was used to select the optimal cutoff point.

Missing data were imputed using the multivariate imputation package (“Chained Equations”) whenever necessary (*n* = 5) (10, 11). A bilateral *p* < 0.05 was considered statistically significant. Data were analyzed with the Stata 15 statistical package (StataCorp. 2017. Stata Statistical Software: Release 15. College Station, TX: StataCorp LLC).

## Results

### Study Population

Among the 1,831 patients included in Redinscor-II registry, plasma Gal-3 concentrations were assessed in 1,201 (65.6%) cases and these constituted the study population. The mean age in the study population was 72 ± 12 years, with 50% of patients being older than 75 years. Women represented 42.3% of the study population. A total of 368 patients (31%) presented left ventricular systolic dysfunction (left ventricular ejection fraction <40%). The index admission was the first episode of AHF for 40.5% of the patients (*de novo* HF), being the remaining 59.5% acute decompensations in chronic heart failure patients (Table 1).

**Table 1:** Baseline characteristic according Gal-3 concentrations above or below median.

	**Total (*N* = 1,201)**	**Gal-3 < 23.2 ng/mL** **(*N* = 601)**	**Gal-3 ≥ 23.2 ng/mL (*N* = 600)**	**Valor p**
**Age, years**	72 ± 12	71 ± 13	74 ± 11	<0.001
**Male**, ***n*** **(%)**	692 (57.6)	363 (60.4)	329 (54.8)	0.054
**Previous HF diagnosis**, ***n*** **(%)**	715 (59.5)	325 (54.1)	390 (65)	0.003
**Systolic pressure, mmHg**	136 ± 29	136 ± 29	132 ± 30	0.029
**Heart rate, bpm**	89 ± 27	91 ± 27	87 ± 26	0.038
**LVEF (%)**				0.79
<40%, *n* (%)	368 (31)	189 (31.4)	179 (29.8)	
40–50%, *n* (%)	185 (15.4)	96 (16)	89 (14.8)	
≥50%, *n* (%)	648 (53.9)	316 (52.6)	332 (55.3)	
**Comorbidities**				
Hypertension, *n* (%)	922 (76.7)	429 (71.4)	493 (82.2)	<0.001
Diabetes mellitus, *n* (%)	557 (46.3)	248 (41.3)	309 (51.5)	<0.001
Dyslipemia, *n* (%)	675 (56.2)	310 (51.6)	365 (60.8)	0.005
eGFR (CKD_EPI) <60 ml/min/1.73 m^2^, *n* (%)	629 (52.4)	204 (33.9)	425 (70.8)	<0.001
Atrial fIbrillation, *n* (%)	448 (37.3)	214 (35.6)	234 (39)	0.28
Ischemic heart disease, *n* (%)	405 (33.7)	187 (31.1)	218 (36.3)	0.31
**Laboratory parameters**				
Hemoglobin, g/dL	12.3 ± 2	12.7 ± 2	1.19 ± 2	<0.001
Sodium, mmol/L	139 ± 5	139 ± 5	138 ± 5	0.097
Potassium, mmol/L	4.3 ± 0.7	4.2 ± 0.6	4.4 ± 0.8	0.004
Creatinin, mg/dL	1.1 ± 0.6	0.9 ± 0.4	1.3 ± 0.6	<0.001
eGFR, (CKD-EPI), mL/min/1.73 m^2^	60 ± 25	70 ± 23	50 ± 23	<0.001
NT-ProBNP, ng/L	3,949 (1,935–8,219)	3,201 (1,550–6,157)	5,336 (2,574–10,113)	<0.001
**Treatment at admission**				
ß-blockers, *n* (%)	674 (56)	325 (54.1)	354 (59)	0.14
ACEI/ARB, *n* (%)	729 (60.7)	358 (59.6)	371 (61.8)	0.44
MRA, *n* (%)	283 (23.5)	120 (20)	169 (28.2)	<0.001
Diuretics, *n* (%)	713 (59.4)	309 (51.4)	404 (67.3)	<0.001
**Treatment at discharge**				
ß-blockers, *n* (%)	835 (69.5)	445 (74)	390 (65)	0.005
ACEI/ARB, *n* (%)	803 (66.9)	434 (72.2)	369 (61.5)	<0.001
MRA, *n* (%)	509 (42.4)	264 (43.9)	245 (40.8)	0.6
Diuretics, *n* (%)	994 (82.8)	498 (82.9)	496 (82.7)	0.37

*ACE, angiotensin–angiotensin-converting enzyme inhibitors; ARB, angiotensin receptor blockers; CKD-EPI, chronic kidney disease epidemiology collaboration; eGFR, estimated glomerular filtration rate; HF, heart failure; LVEF, left ventricular ejection fraction; MRA, mineralocorticoids receptors antagonists; NT-proBNP, aminoterminal portion of the proBNP molecule*.

**Figure 1:**
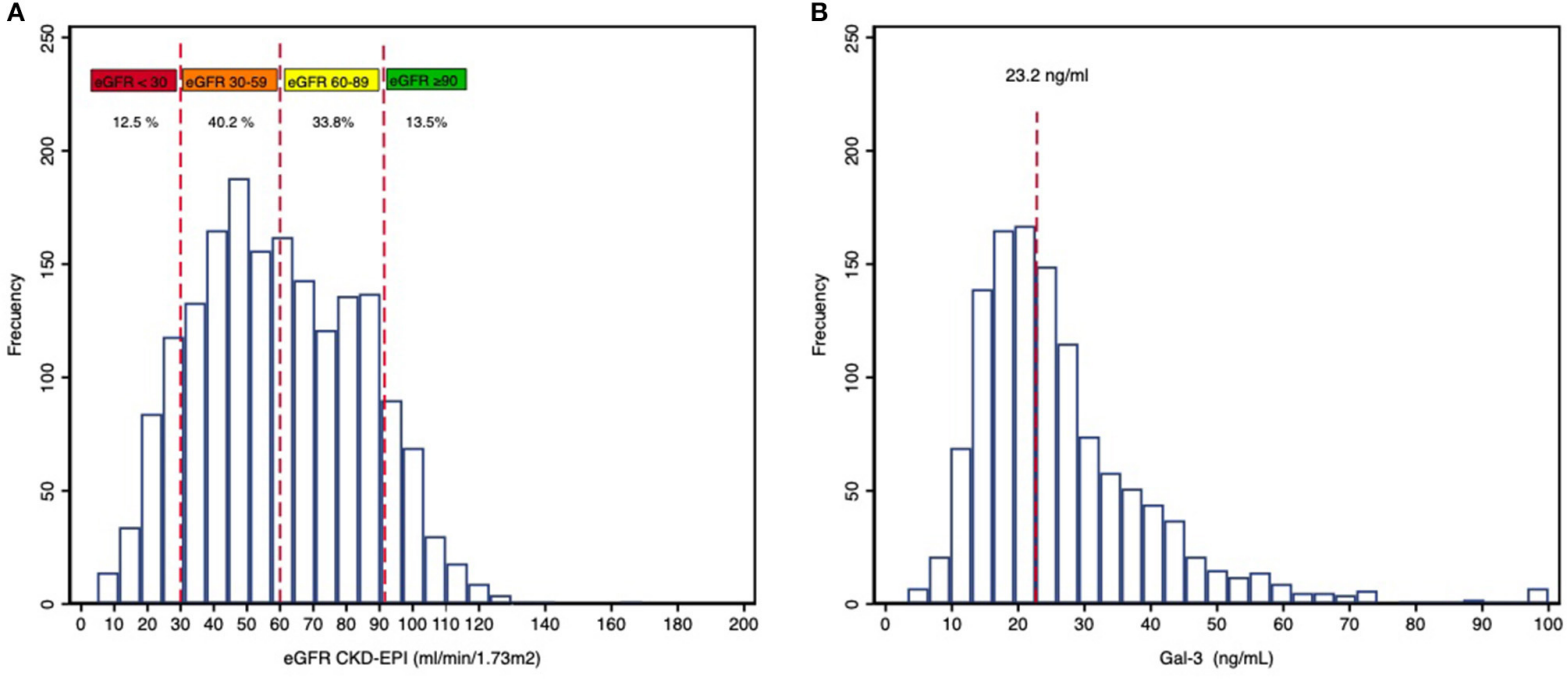
Distribution of eGFR **(A)** and Gal-3 **(B)** values in the population.

Mean eGFR at admission was 60 ± 25 ml/min/1.73 m^2^, with 52.7% of patients with GFR < 60 ml/min/1.73 m^2^ (Figure 1A) illustrates the distribution of eGFR values in the study population.

Median serum Gal-3 concentrations at admission were 23.2 (17.3–32.1) ng/mL (Figure 1B). Patients with elevated Gal-3 concentrations presented a greater burden of comorbidities, with a higher prevalence of hypertension, diabetes, dyslipidemia, and parameters of greater clinical severity with higher concentrations of serum potassium and NT-proBNP. These patients also presented lower hemoglobin concentrations, worse eGFR, and a lower blood pressure on admission (Table 1).

### Interaction Between Renal Function and Gal-3 Concentrations

**Figure 2:**
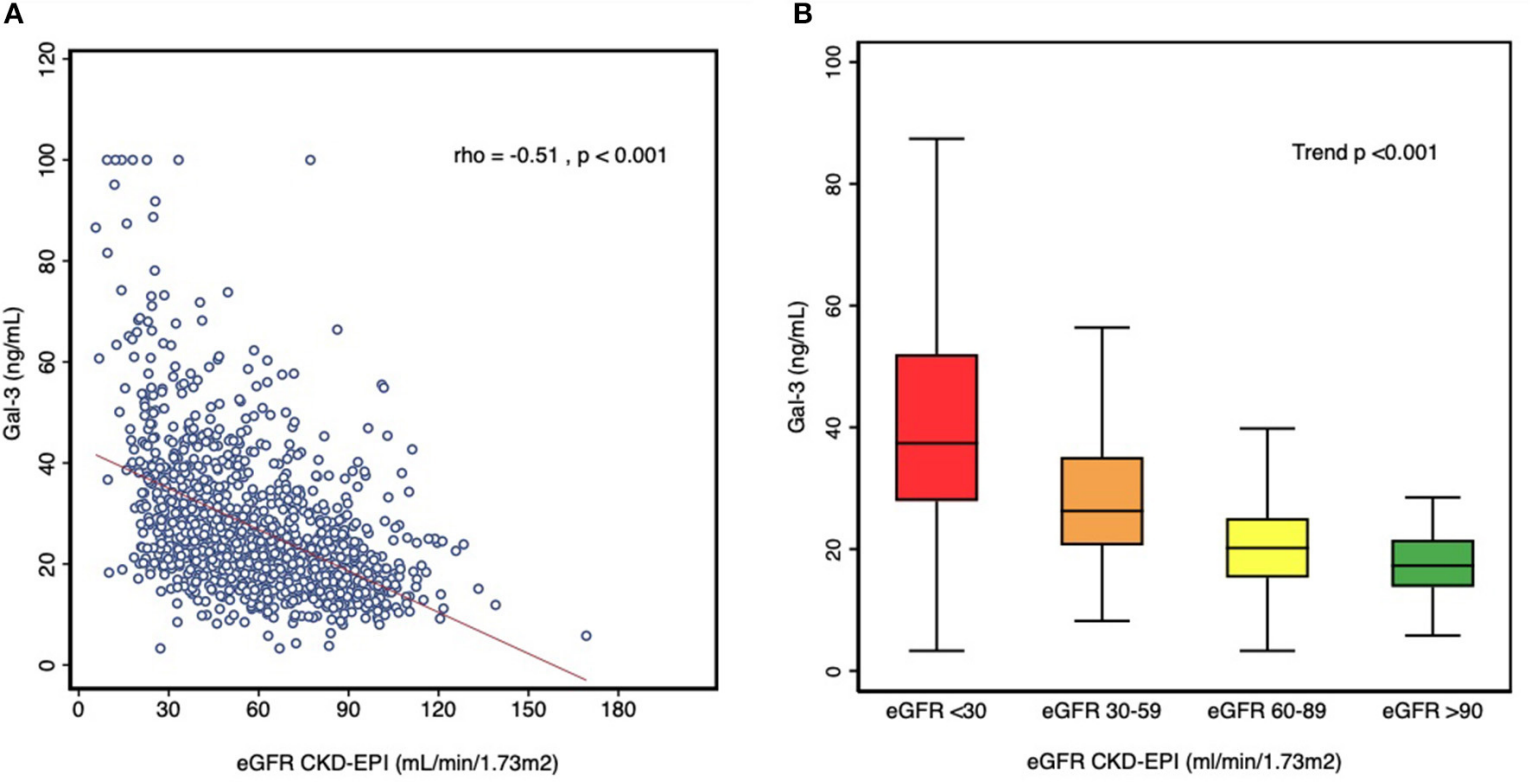
**(A)** Correlation between Gal-3 levels and eGFR. **(B)** Box plot of Gal-3 levels across stages of renal dysfunction.

Gal-3 concentrations showed significant negative correlation with eGFR at admission (Spearman correlation coefficient, rho = −0.51; *p* < 0.001) (Figure 2A) with a significant trend (*p* < 0.001) to increase with eGFR worsening (Figure 2B).

The median Gal-3 concentration was 19 (14.8–24.6) ng/mL, for patients with eGFR ≥ 60 ml/min/1.73 m^2^; and 28.2 (20.6–35.1) ng/ml for those with GFR <60 ml/min/1.73 m^2^. Table 2 shows the characteristics of the population according to the presence or not of renal dysfunction and the Gal-3 values above or below the median for each group. In both eGFR groups, patients with elevated Gal-3 concentrations presented parameters suggesting a greater clinical severity, with lower serum hemoglobin concentrations, eGFR and higher NT-proBNP values.

**Table 2:** Baseline characteristic according to groups of renal dysfunction and Gal-3 levels above or below median.

	**eGFR ≥60 ml/min/1.73 m** ^**2**^			**eGFR < 60 ml/min/1.73 m** ^**2**^		
	**Gal-3 ≤19 ng/mL** **(*N* = 289)**	**Gal-3 > 19 ng/mL** **(*N* = 283)**	***p-*value**	**Gal-3 ≤28.2 ng/mL** **(*N* = 315)**	**Gal-3 > 28.2 ng/mL** **(*N* = 314)**	***p-*value**
**Age, years**	67 ± 14	70 ± 13	0.004	76 ± 10	75 ± 9	0.38
**Male**, ***n*** **(%)**	180 (62.3)	162 (57.2)	0.23	168 (53.3)	182 (58)	0.26
**Previous HF diagnosis**, ***n*** **(%)**	139 (48.1)	140 (49.5)	0.95	215 (68.3)	221 (70.4)	0.56
**Systolic pressure, mmHg**	137 ± 28	135 ± 28	0.44	135 ± 30	131 ± 31	0.11
**Heart rate, bpm**	93 ± 26	93 ± 28	0.90	86 ± 28	85 ± 23	0.49
**LVEF (%)**			0.19			0.74
<40%, *n* (%)	94 (32.5)	90 (31.8)		90 (28.6)	94 (29.9)	
40–50%, *n* (%)	56 (19.4)	36 (12.7)		49 (15.6)	44 (14)	
≥50%, *n* (%)	139 (48.1)	157 (55.5)		176 (55.9)	176 (56.1)	
**Comorbidities**						
Hypertension, *n* (%)	186 (64.4)	202 (71.4)	0.074	263 (83.5)	271 (86.3)	0.59
Diabetes mellitus, *n* (%)	109 (37.7)	104 (36.7)	0.97	161 (51.1)	183 (58.3)	0.19
Dyslipemia, *n* (%)	144 (49.8)	144 (50.9)	0.35	187 (59.4)	200 (63.7)	0.17
Atrial fibrillation, *n* (%)	77 (26.6)	77 (27.2)	0.85	117 (37.1)	134 (42.7)	0.27
**Laboratory parameters**						
Hemoglobin, g/dL	13.1 ± 1.8	12.6 ± 2.1	0.009	12.1 ± 1.8	1.16 ± 2	<0.001
Sodium, mmol/L	139 ± 4	138 ± 5	0.019	138 ± 5	138 ± 5	0.87
Potassium, mmol/L	4.1 ± 0.6	4.2 ± 0.6	0.29	4.5 ± 0.7	4.4 ± 0.8	0.36
Creatinin, mg/dL	0.75 ± 0.2	0.77 ± 0.2	<0.001	1.27 ± 0.4	1.63 ± 0.7	<0.001
eGFR, (CKD-EPI), mL/min/1.73m^2^	84 ± 16	79 ± 14	<0.001	44 ± 10	36 ± 12	<0.001
NT-ProBNP, ng/L	2,646 (1,246–4,256)	4,040 (2,299–7,163)	<0.001	4,709 (2,236–8,055)	7,097 (2,617–11,722)	<0.001
**Treatment at admission**						
ß-blockers, *n* (%)	145 (50.2)	148 (52.3)	0.60	190 (60.3)	191 (60.8)	0.96
ACEI/ARB, *n* (%)	160 (55.4)	164 (58.0)	0.56	208 (66)	197 (62.7)	0.41
MRA, *n* (%)	38 (13.1)	59 (20.8)	0.018	97 (30.8)	89 (28.3)	0.44
Diuretics, *n* (%)	169 (58.5)	155 (54.8)	0.28	187 (59.4)	202 (64.3)	0.29
**Treatment at discharge**						
ß-blockers, *n* (%)	221 (76.4)	204 (72.1)	0.29	218 (69.2)	192 (61.1)	0.10
ACEI/ARB, *n* (%)	218 (75.4)	204 (72.1)	0.58	207 (65.7)	174 (55.4)	0.02
MRA, *n* (%)	128 (44.3)	134 (47.3)	0.35	136 (43.2)	111 (35.4)	0.08
Diuretics, n (%)	237 (82)	231 (81.6)	0.8	269 (85.4)	257 (81.8)	0.61

*ACE, angiotensin–angiotensin-converting enzyme inhibitors; ARB, angiotensin receptor blockers; CKD-EPI, chronic kidney disease epidemiology collaboration; eGFR, estimated glomerular filtration rate; HF, heart failure; LVEF, left ventricular ejection fraction; MRA, mineralocorticoids receptors antagonists; NT-proBNP, aminoterminal portion of the proBNP molecule*.

In multivariate linear regression analysis, Gal-3 levels were independently predicted by age, eGFR, hemoglobin concentrations and NT-proBNP (Table 3).

**Table 3:** Univariate and multivariate analysis of Gal-3 predictors.

	**Univariate**		**Multivariate**	
	***b* (95%-CI)**	***p-*value**	***b* (95%-CI)**	***p-*value**
eGFR (CKD-EPI)	−0.009 (−0.010, −0.008)	<0.001	−0.008 (−0.009, 0.007)	<0.001
Age, years	0.007 (0.004–0.009)	<0.001		
Sex (male)	0.002 (−0.057–0.051)	0.915		
Previous HF diagnosis	0.102 (0.047–0.157)	<0.001		
Hypertension	0.149 (0.085–0.212)	<0.001		
Diabetes mellitus	0.105 (0.051–0.158)	<0.001		
Systolic pressure	−0.001 (−0.002, −0.001)	0.032		
Hemoglobin	−0.005 (−0.006, −0.004)	<0.001	−0.002 (−0.003, −0.001)	<0.001
Sodium	−0.005 (−0.011, −0.000)	0.057		
Potassium	0.076 (0.038–0.115)	<0.001		
NT-ProBNP	0.002 (0.001–0.002)	<0.001	0.001 (0.000–0.001)	<0.001

*eGFR, estimated glomerular filtration rate by CKD-EPI chronic kidney disease epidemiology collaboration formula; HF, heart failure; NT-proBNP, aminoterminal portion of the proBNP molecule*.

### Prognostic Value of Gal-3 in Relation to Renal Function

A total of 236 patients (19.7%) died during the 12-month follow-up. Patients with Gal-3 concentrations above the median (23.2 ng/mL) had a higher mortality rate than those with concentrations below the median value (24.8 vs. 14.5%; *p* < 0.001). The main cause of mortality was due to cardiovascular causes, but also to an increase in mortality from non-cardiovascular. In renal dysfunction group, the increased mortality observed in patients with Gal 3 above median (>28.2 ng/mL) was driven by an increase in mortality from non-cardiovascular causes (Table 4).

**Table 4:** Mortality and cause of death according to groups of renal dysfunction and Gal-3 levels above or below median.

	**Gal-3 < 23.2 ng/mL (*****N*** **= 601)**			**Gal-3 ≥ 23.2 ng/mL (*****N*** **= 600)**		***p-*value**
In-hospital mortality, *n* (%)	12 (2)			29 (4.8)		0.026
12-month all-cause mortality, *n* (%)	87 (14.5)			149 (24.8)		<0.001
Cardiovascular death, *n* (%)	66 (11)			110 (18.3)		<0.001
Sudden death, *n* (%)	19 (3.2)			22 (3.7)		0.63
Heart failure death, *n* (%)	40 (6.7)			66 (11)		0.008
Non-cardiovascular death, *n* (%)	21 (3.5)			39 (6.5)		0.017
	**eGFR ≥ 60 ml/min/1.73 m** ^**2**^			**eGFR < 60 ml/min/1.73 m** ^**2**^		
	**Gal-3 ≤ 19 ng/mL** **(*****N*** **= 289)**	**Gal-3 >19 ng/mL** **(*****N*** **= 28)**	***p-*** **value**	**Gal-3 ≤ 28.2 ng/mL** **(*****N*** **= 315)**	**Gal-3 > 28.2 ng/mL** **(*****N*** **= 314)**	***p-*** **value**
In-hospital mortality, *n* (%)	3 (1)	7 (2.5)	0.33	10 (3.2)	21 (6.7)	0.091
12-month all-cause mortality, *n* (%)	30 (10.4)	34 (12)	0.54	70 (22.2)	102 (32.5)	0.004
Cardiovascular death, *n* (%)	21 (7.3)	22 (7.8)	0.82	58 (18.4)	75 (23.9)	0.093
Sudden death, *n* (%)	10 (3.5)	5 (1.8)	0.21	12 (3.8)	14 (4.5)	0.68
Heart failure death, *n* (%)	10 (3.5)	13 (4.6)	0.49	38 (12.1)	45 (14.3)	0.40
Non-cardiovascular death, *n* (%)	9 (3.1)	12 (4.2)	0.47	12 (3.8)	27 (8.6)	0.013

*eGFR, estimated glomerular filtration rate by CKD-EPI chronic kidney disease epidemiology collaboration formula*.

**Figure 3:**
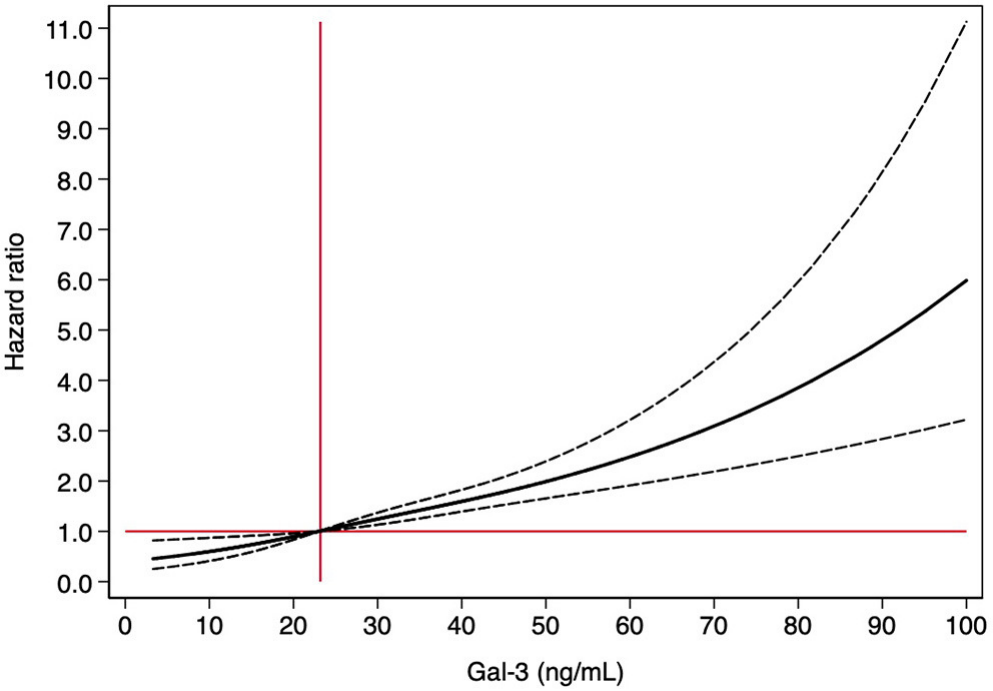
Relationship between Gal-3 levels and 12-month mortality. The figure shows the hazard ratio, with 95% confidence intervals, for Gal-3 using Cox regression analysis. Gal-3 were modeled with cubic splines with three knots. The median levels of Gal-3 (23.2 ng/ml, vertical line) was fixed as reference level in the total population.

In the univariate analysis, Gal-3 concentrations (ng/mL) were associated with a higher risk of all-cause mortality [HR: 1.025 (95%-CI: 1.018–1.032); *p* < 0.001]. In the multivariate analysis, after adjusting for eGFR and other prognostic variables, Gal-3 maintained its independent association with mortality [HR: 1.010 (95% CI: 1.001–1.018); *p* = 0.038] (Table 5). No significant interaction between Gal-3 and renal function was observed (*p* = 0.235). The relationship of Gal-3 with mortality draws an exponential hazard curve, with a continuous growth of mortality risk as Gal-3 values increased (Figure 3). The multivariate Cox model had a Harrell's C-statistic of 0.71, and the Gronnesby and Borgan goodness-of-fit test showed good model calibration (*p*-value > 0.10).

**Table 5:** Univariate and multivariate analysis of 12 months mortality.

	**Univariate**		**Multivariate**	
	**HR (95%-CI)**	***p-*value**	**HR (95%-CI)**	***p-*value**
**Total population**				
Galectin-3	1.025 (1.018–1.32)	<0.001	1.010 (1.001–1.018)	0.038
Age	1.042 (1.027–1.057)	<0.001	1.035 (1.019–1.050)	<0.001
Diabetes mellitus	1.265 (0.997–1.606)	0.053		
Functional class (NYHA)	1.891 (1.581–2.261)	<0.001	1.633 (1.318–2.100)	<0.001
Systolic pressure	0.992 (0.988–0.996)	<0.001	0.994 (0.989–0.999)	0.035
Residual congestion	1.156 (1.056–1.265)	0.002		
Hemoglobin	0.986 (0.974–0.999)	0.038		
eGFR (CKD_EPI)	0.986 (0.979–0.991)	<0.001	0.989 (0.982–0.997)	0.043
Sodium	0.960 (0.937–0.984)	0.001		
Ln NT-ProBNP	1.608 (1.452–1.781)	<0.001	1.333 (1.162–1.530)	<0.001
ACEI o ARB	0.499 (0.379–0.657)	<0.001	0.637 (0.480–0.844)	0.002
ß-blockers	0.707 (0.558–0.895)	0.004		
**eGFR ≥ 60 ml/min/1.73 m** ^**2**^				
Gal-3	1.012 (0.989–1.035)	0.303	0.990 (0.964–1.017)	0.472
Gal-3 > 19.7 ng/mL	1.188 (0.727–1.941)	0.491	0.728 (0.425–1.248)	0.250
**eGFR < 60 ml/min/1.73 m** ^**2**^				
Gal-3	1.017 (1.008–1.026)	<0.001	1.010 (1.001–1.019)	0.033
Gal-3 > 31.5 ng/mL	1.942 (1.405–2.685)	<0.001	1.560 (1.126–2.162)	0.007

*The covariates evaluated were age, diabetes mellitus, previous HF diagnosis, systolic blood pressure, hemoglobin, natremia, eGFR, LVEF, residual congestion, NYHA functional class, and treatment with RAAS inhibitors, beta blockers and diuretics*.

Afterwards, we evaluated the prognostic performance of Gal-3 concentrations for the different eGFR subgroups. In patients with significant renal dysfunction (eGFR < 60 mL/min/1.73 m^2^), Gal-3 remained an independent predictor of mortality in multivariate analysis [HR: 1.010 (1.001–1.019); *p* = 0.033]. In contrast, in those patients with preserved renal function (eGFR ≥ 60 mL/min/1.73 m^2^), Gal-3 was not associated with increased mortality risk [HR: 0.990 (0.964–1.017); *p* = 0.472].

**Figure 4:**
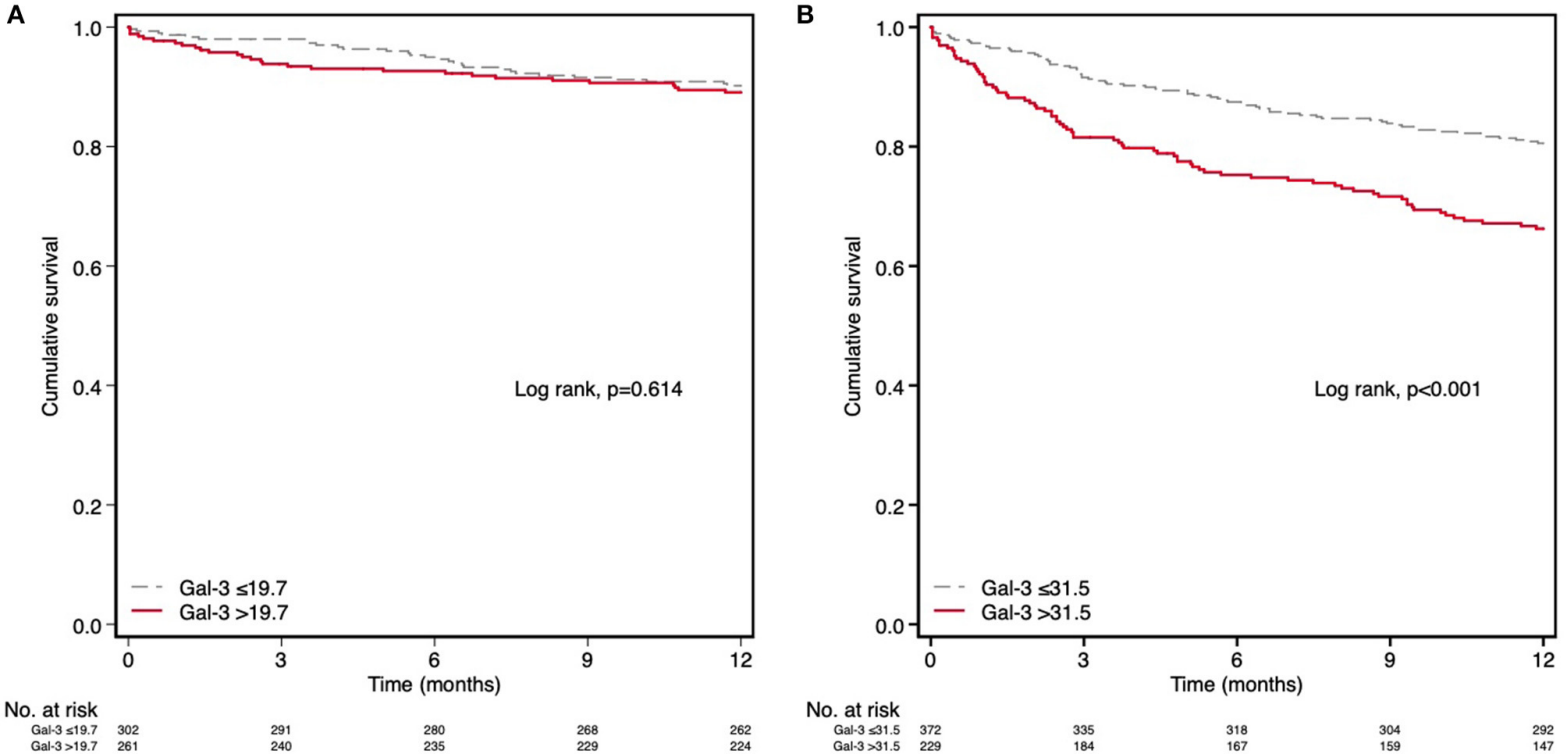
Kaplan–Meier survival analysis for 12-month all-cause mortality according to the optimal cutoff point of Gal-3 in the different groups of eGFR. **(A)** Preserved renal function (eGFR ≥60 ml/min/1.73 m^2^) and Gal-3 > 19.7 ng/mL. **(B)** Renal dysfunction (eGFR <60 ml/min/1.73 m^2^) and Gal-3 > 31.5 ng/mL.

We analyzed ROC curves to determine whether there was an optimal Gal-3 concentration for predicting mortality in the two renal function groups. For patients with preserved renal function, a 19.7 ng/mL cutoff point had a sensitivity and specificity of 51.6 and 53.5%, respectively. The optimal cutoff point in those with impaired renal function was 31.5 ng/mL (sensitivity 54%, specificity 65.9%). In the multivariate analysis of the latter group, the concentration of 31.5 ng/mL was significantly associated with higher mortality [HR: 1.560 (1.126–2.162); *p* = 0.007]; in contrast, in patients with preserved renal function, the Gal-3 concentration of 19.7 ng/mL was not associated with higher mortality [HR: 0.728 (0.425–1.248); *p* = 0.250] (Table 5). Figure 4 shows the Kaplan-Meier curves of 12-month all-cause mortality in each renal function group according to the different Gal-3 cutoff points.

## Discussion

**Figure 5:**
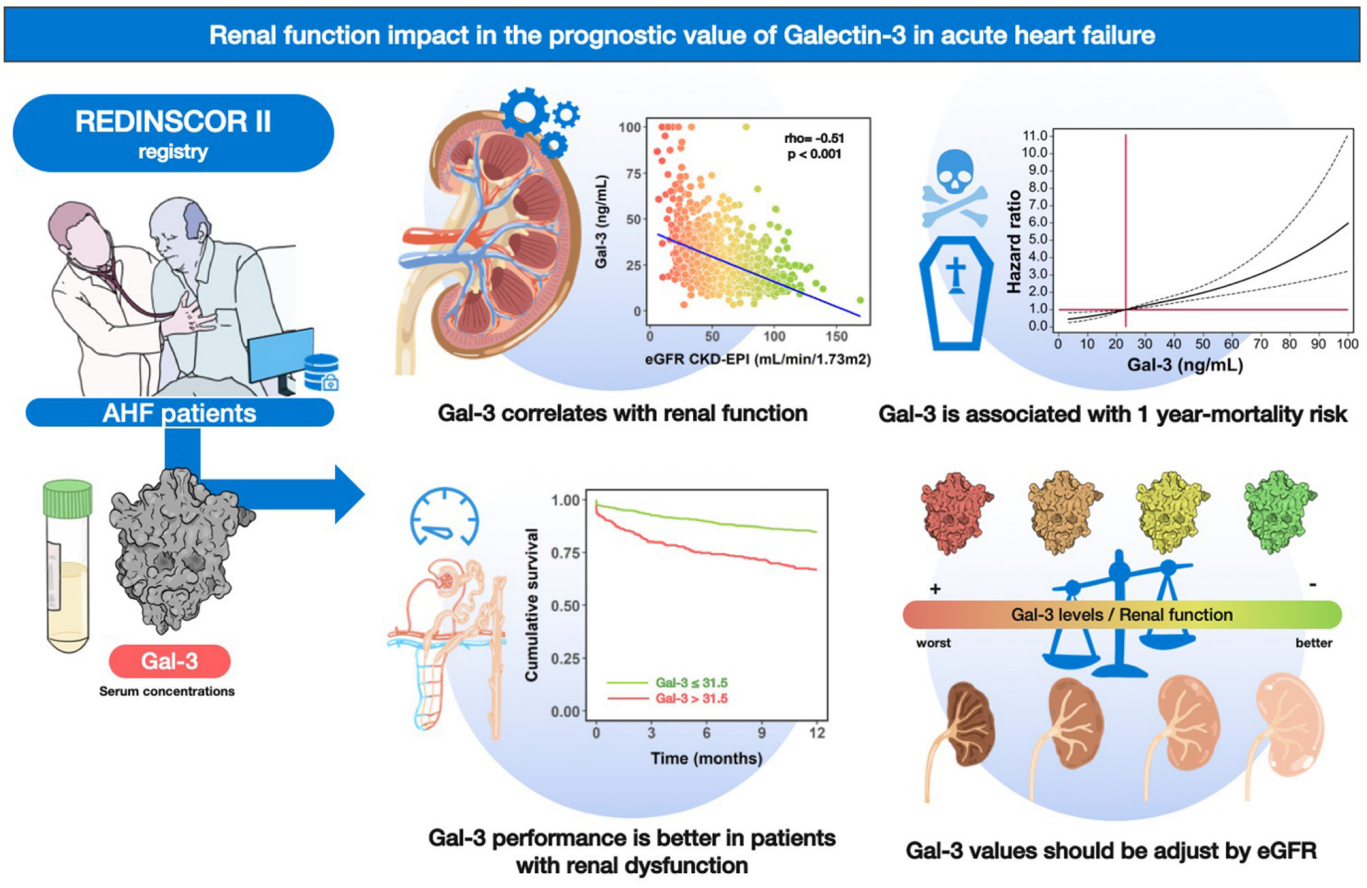
Graphical abstract. Gal-3 and renal function in patients with acute heart failure: relations and prognosis.

In this subanalysis of the Redinscor-II registry, we evaluated the prognostic value of Gal-3 in relation to the renal function in patients admitted to the hospital for an episode of AHF. The main findings of this study are: (1) there is a negative correlation between Gal-3 and renal function; (2) Gal-3 concentrations are associated with an increased risk of mortality during 1-year follow-up, and its prognostic value is independent of eGFR; (3) However, Gal-3 concentrations loses their predictive value in those patients with eGFR ≥ 60 mL/min/1.7 m^2^; (4) the optimal Gal-3 cutoff points for predicting mortality vary according to renal function, suggesting that a reliable interpretation of Gal-3 requires adjustment for eGFR values (Figure 5).

Overall, our results indicate that Gal-3 is a valuable biomarker in assessing cardiorenal interaction during an episode of AHF. Despite its dependence on renal function, Gal-3 provides prognostic and independent information, being effective in stratifying mortality risk in patients with AHF.

### Gal-3 and Renal Function in AHF

Gal-3 shows a negative correlation with renal function in patients with AHF, such that the more significant the deterioration in eGFR, the higher the Gal-3 concentration. This finding has already been observed in previous smaller studies on HF patients (7, 12–14), but few have delved into the impact that renal function may have on the prognostic value of Gal-3. Thus, a strong correlation between Gal-3 and renal function (*r* = −0.77; *p* < 0.001) has been already described, but any association with the severity of the AHF episode nor the timing of HF (acute or chronic) (14). Patients with HF and renal dysfunction present a lower renal clearance of Gal-3, which cause an increase in plasmatic concentrations (15). In addition, in animal models of renal injury induced by episodes of ischemia-reperfusion, an increased renal production of Gal-3 has been observed as a response to tubular damage (16).

### Impact of Renal Function on the Prognostic Value of Gal-3

Gal-3 is an inflammatory biomarker secreted by activated macrophages that promotes fibrosis in different organs, including the heart and kidney (5, 17, 18). Numerous investigations in HF have confirmed that elevated plasma Gal-3 contributes to the progression of the disease and increases the risk of adverse events, with a higher risk of mortality and hospitalization during follow-up (19, 20).

In our population, the prognostic value of Gal-3 was maintained after adjusting for renal function (eGFR) and other prognostic variables in the multivariate analysis; this fact is of particular interest for evaluating cardiorenal interaction during an episode of AHF. However, when patients with and without renal dysfunction were evaluated separately, we observe that Gal-3 loses its prognostic value in patients with preserved renal function. For this reason, the optimal Gal-3 cutoff points for predicting mortality vary according to eGFR. Thus, in patients with eGFR ≥ 60 mL/min, the optimal cutoff point is a Gal-3 concentration of 19.7 ng/mL, whereas in patients with GFR < 60 mL/min/1.73 m^2^ is of 31.5 ng/mL, highlighting the importance of selecting Gal-3 values according to the existence of renal failure to improve the performance of this biomarker. Overall, our work comprehensively exposes the influence of renal function on the prognostic value of Gal-3 and guides the correct interpretation of its values within the spectrum of renal disease.

To date, few studies have evaluated the impact that renal function has on the prognostic value of Gal-3 among HF patients, and they show contradictory results (7, 13). Zhang et al. (7) evaluated the prognostic performance of Gal-3 in 1,161 patients hospitalized for AHF in a single study. Their results show that Gal-3 is an independent marker of mortality, but unlike our work, mortality risk was not significant in patients with an eGFR lower than 60 mL/min/1.73 m^2^. This discrepancy may be explained by the heterogeneity of the population included in this study and by its lower risk profile. To summarize, this study included younger patients; with a lower burden of comorbidities; lower NT-proBNP concentrations, with median NT-proBNP of 1,489 ng/mL compared to 3,949 ng/mL in our work; and better renal function, with only 28% of the patients with eGFR < 60 ml/min/1.73 m^2^ against 52% in our population. Additionally, a factor that can promote heterogeneity among Galectin-3 results of different studies is the method used for its measurement. Up to date, Galectin-3 can be measured by several methods based on different principles and using different antibodies to identify the molecule. Indeed, there not exist a standard that allows to homogenize results of different assays. Thus, methodology plays a role on the discrepancies among studies. In a study published by Zamora et al. (13), including outpatients with HF with a follow-up of 4 years, Gal-3 lost its prognostic value when renal function was included in the multivariate model. However, it is interesting to note that, similar to our results, the study observed a different predictive capacity of Gal-3 according to the stratum of renal disease, with greater discriminative power in patients with an eGFR between 30 and 60 mL/min/1.73 m^2^.

The pathophysiological mechanisms explaining the prognostic value of Gal-3 in the setting of HF have not been fully characterized, but it is known that Gal-3 concentrations are strongly associated with the degree of neurohumoral activation (12, 21) and with the reduction in renal function (14). However, an association between Gal-3 and cardiac function parameters or clinical severity has not been described, which forces us to question whether the cardiotoxic effect of Gal-3 is the only determinant of its predictive value. Gal-3 is a pleiotropic molecule that reflects inflammation and fibrosis in relation to tissue damage, and it is not exclusive to a single organ. The close influence of renal function on the prognostic value of Gal-3 suggests that worsening HF and the risk of adverse events may also be mediated by worsening renal function. Iacoviello et al. (22) observed in 260 patients with chronic HF that elevated Gal-3 concentrations were associated with a progressive deterioration of eGFR during 1-year follow-up. This evidence supports the hypothesis that Gal-3 would reflect not only the progression of cardiac dysfunction but also renal deterioration. Our work shows that patients with high Gal-3 levels and renal dysfunction have an increased risk for non-cardiovascular mortality. We ignore whether the reason for this increased risk of non-cardiovascular mortality was due to a progression of kidney disease.

### Potential Applications of Gal-3 in the Cardiorenal Syndrome

Cardiorenal syndrome defines the spectrum of alterations that affect the heart and kidney due to the deleterious interaction between both organs through hemodynamic, neurohormonal and inflammatory mechanisms (23). In HF, it is especially important due to the high prevalence of renal dysfunction and the impact on adverse events (24). In this scenario, the quantification of different biomarkers assessing different physiopathologic aspects (multimarker approach) might improve diagnostic efficiency and risk stratification (21). Recently, Zannad and Rossignol (6) have published a white paper on cardiorenal syndrome as a future guide for its approach. The authors propose a phenotype-biomarker approach with a particular interest in the role of inflammation and fibrosis. Our work aims to support this approach since Gal-3 identifies a patient profile with increased inflammatory activity and shows good prognostic performance in subjects with HF and impaired renal function.

It is still unknown whether Gal-3 is only a risk marker reflecting the inflammatory status or the direct cause of a worse prognosis. Experimental work in animal models has demonstrated the protective effect of Gal-3 inhibition on cardiac and renal damage (25). In this sense, Gal-3 could be considered as a potential therapeutic target to develop specific pharmacological interventions.

### Study Limitations

The Redinscor-II registry is a prospective observational registry, which has the limitations inherent to the nature of these epidemiological studies. The findings should not be interpreted as causal/confirmatory but as hypothesis-generating. The analyses were performed adjusting for the main confounding variables; we are unaware of the influence of possible factors that were not considered.

## Conclusions

In patients with AHF, Gal-3 concentrations negatively correlate with renal function. Gal-3 concentrations are associated with an increased risk of 1-year mortality independently of eGFR, although its discriminative power disappears in patients with eGFR ≥ 60 mL/min/1.73 m^2^.

The optimal cutoff points for predicting a higher mortality risk are varies depending on eGFR. For that reason, for the correct prognostic interpretation of Gal-3 values, they should be stratified by renal function.

## Data Availability Statement

The raw data supporting the conclusions of this article will be made available by the authors, without undue reservation.

## Ethics Statement

The studies involving human participants were reviewed and approved by Comité ética Hospital 12 Octubre. The patients/participants provided their written informed consent to participate in this study.

## Author Contributions

All authors listed have made a substantial, direct, and intellectual contribution to the work and approved it for publication.

## Funding

This work was supported by grants from Redes Temáticas de Investigación Cooperativa en Salud del Instituto de Salud Carlos III (REDINSCOR), Madrid, Spain (Grant No. RD06-0003-0000) and Red de Investigación Cardiovascular del Instituto de Salud Carlos III (RIC), Madrid, Spain (Grant No. RD12/0042/0002).

## Conflict of Interest

The authors declare that the research was conducted in the absence of any commercial or financial relationships that could be construed as a potential conflict of interest.

## Publisher's Note

All claims expressed in this article are solely those of the authors and do not necessarily represent those of their affiliated organizations, or those of the publisher, the editors and the reviewers. Any product that may be evaluated in this article, or claim that may be made by its manufacturer, is not guaranteed or endorsed by the publisher.

## Abbreviations

AHF: acute heart failure
CKD: chronic kidney disease
CKD-EPI: chronic kidney disease epidemiology collaboration
eGFR: estimated glomerular filtration rate
Gal-3: Galectin-3
HF: heart failure
LVEF: left ventricular ejection fraction
NT-proBNP: aminoterminal portion of the proBNP molecule
ROC: receiver operating characteristic curve.
